# Unrecognized mutations in DPYD* 2 A wild-type rectal cancer patients receiving postoperative 5-FU-based chemotherapy - do they have a clinical impact?

**DOI:** 10.1007/s00280-025-04787-4

**Published:** 2025-07-15

**Authors:** P. Liersch, S. Dierks, R. Andag, T. Liersch, C. de Boer, J. Kreutzer, A. Hille, H. Sülberg, A. Leha, Julie Schanz

**Affiliations:** 1 Department of Haematology and Medical Oncology, University Medical Center of the Georg August University, Göttingen, Germany; 2 Department of Oral Surgery and Implantology, Carolinum, University Medical Center of the Johann Wolfgang von Goethe University, Frankfurt am Main, Germany; 3 Department of Clinical Chemistry, University Medical Center of the Georg August University, Göttingen, Germany; 4 Department of General, Visceral and Paediatric Surgery, University Medical Center of the Georg August University, Göttingen, Germany; 5 Department of Internal Medicine, Städtisches Klinikum Kiel, Kiel, Germany; 6 Department of Radiotherapy and Radiation Oncology, University Medical Center of the Georg August University, Göttingen, Germany; 7 X-act Cologne Clinical Research GmbH, Cologne, Germany; 8 Department of Medical Statistics, University Medical Center of the Georg August University, Göttingen, Germany; 9 Interdisciplinary UMG laboratories, Department of Clinical Chemistry, University Medical Center of the Georg August University, Göttingen, Germany

**Keywords:** 5-Fluorouracil, Dihydropyrimidine-dehydrogenase, *DPYD*, Genotyping, Pharmacogenetics, Rectal cancer

## Abstract

**Purpose:**

The impact of the unrecognized mutational dihydropyrimidine-dehydrogenase-gene-(*DPYD*)*-*status on high-grade CTC-AE-grades ≥ 3 (NCI-Common Terminology Criteria for Adverse Events, vs. 3.0) was assessed in patients with upper rectal cancer (inferior tumor margin ≥ 12 cm above the anal verge) treated with upfront surgery and 5-Fluorouracil (5-FU) based adjuvant chemotherapy (CTx).

**Methods:**

75 participants of the GAST-05-phase-IIb-trial (ISRCTN35198481) were tested in this single center analysis for DPYD*2A-wildtype (WT) at staging. After surgery, 43 patients (stages II and III, according to the current 8th TNM/UICC-classification, 2017) received FOLFOX-CTx and entered follow-up (median: 101 months). According to recent recommendations of the European Medicines Agency (EMA) and national guidelines, post-hoc genotyping for DPYD*2A (c.1905 + 1G > A; IVS14 + 1G > A; rs3918290), DPYD*13 (c.1679T > G; rs55886062), polymorphism c.2846 A > T (rs67376798) and Haplotype B3 (HapB3) (c.1236G > A; c.1129–5923 C > G) was performed using cryopreserved blood samples and standardized PCR-techniques.

**Results:**

Five patients were found to have a heterozygous (het_) *DPYD*-HapB3-status. Across all patients, the adherence to CTx-cycles 1 to 4 was 100%, 97.7%, 95.3%, and 93.0%, respectively. Grade ≥ 3 CTC-AEs were observed in 0.9% of both het_HapB3- and WT-patients. The mean administered dose of 5-FU was 68.8% of the target in *DPYD*-HapB3 carriers, compared to 92.6% in 38 WT patients. Logistic regression analysis revealed that 5-FU dose reductions were significantly associated with *DPYD*-HapB3 carrier status (odds ratio [OR] 12.55, *p* = 0.044) and male sex (OR 0.23, *p* = 0.049). During follow-up het_HapB3-patients had a recurrence rate of 60.0%, compared to 13,6% for WT-patients. The disease-free survival (DFS) for het_HapB3-patients was significantly reduced vs. WT (*p* = 0.010). Multivariable analysis showed that het_HapB3-patients had an increased risk for reduced DFS (HR 3.774; *p* = 0.057). Interestingly, 5-FU dose reductions per se were not significantly associated with limited DFS in the total population.

**Conclusion:**

*DPYD* genotyping revealed a het_HapB3 variant in 11.6% of DPYD*2A-WT patients treated with FOLFOX. While not linked to increased toxicity, HapB3 status was associated with reduced DFS, suggesting an impact on treatment efficacy. These results support *DPYD* genotyping and highlight the need for adequate 5-FU plasma level assessment followed by subtile dose escalation (therapeutic drug monitoring) to personalize 5-FU dosing more precisely, safely and most effective.

**Supplementary Information:**

The online version contains supplementary material available at 10.1007/s00280-025-04787-4.

## Introduction

5-Fluorouracil (5-FU) is successfully used for pre- and postoperative treatment of patients with solid malignancies worldwide [1–3]. In patients with locally advanced rectal cancer (LARC, stages II or III), 5-FU is well established as sensitizer for preoperative long-term irradiation (RT, 50.4 Gy) alone or in combination with oxaliplatin (OX) as shown in several large randomized trials [4–9]. Additionally, infusions of 5-FU continuously applied at high doses do not only represent the backbone for postoperative chemotherapy (CTx) using FOLFOX [10–12], but also for innovative total neoadjuvant therapy (TNT), such as used in the CAO/ARO/AIO-12-, RAPIDO-, PRODIGE-23-trials [13–15].

However, 5-FU-based CTx regimens can be associated with high grade (G) toxicities G3–G4 [16] in 10–40%, G4 toxicities in 4–9%, and G5 (death) in 0.2–0.5% of patients [17, 18]. The incidence of death, life-threatening prognosis or disability amounts to 1.4% during the first two cycles of fluoropyrimidine-based CTx [19].

Early severe toxicities associated with 5-FU are predominantly caused by impaired pharmacokinetics due to single nucleotide polymorphisms (SNPs) in the dihydropyrimidine-dehydrogenase (DPD) gene (*DPYD*) [20, 21]. Most of these SNPs are responsible for amino acid changes leading to enzymatic dysfunctions. Therefore, homo- or heterozygous (homo/het) mutational *DPYD* status can result in a complete or partial loss of DPD-activity followed by rapid 5-FU accumulation and highly increased toxicity (G ≥ 3).

In April 2020, the European Medicines Agency (EMA) [22] recommended testing for the most common *DPYD*-mutations (DPYD*2A (c.1905 + 1G > A; IVS14 + 1G > A; rs3918290), DPYD*13 (c.1679T > G; rs55886062), polymorphism c.2846 A > T (rs67376798) and Haplotype B3 (HapB3) (c.1236G > A; c.1129–5923 C > G)) in patients prior to 5-FU exposition to identify DPD deficiency.

Evidence for these recommendations have been shown in three separate meta-analyses summarizing data of more than 18.600 patients treated with 5-FU (Table A.1) [23–26].

A central question in the context of any multimodal treatment (MMT) in patients with LARC is whether 5-FU-related toxicity may be attributable to *DPYD* mutations not initially screened for - specifically, those beyond the DPYD*2A variant that are now recommended for routine testing by regulatory bodies such as the EMA and DGHO. In this post-hoc analysis, we assessed the clinical impact of these additional *DPYD*-variants in patients from the GAST-05 phase IIb study (ISRCTN 35198481; Figure A.1), all of whom had been prospectively genotyped and confirmed as DPYD*2A wild-type at study entry. We examined whether the presence of previously undetected variants was associated with toxicity events, CTx administration, 5-FU dose adjustments, and survival.

## Patients, material and methods

**Fig. 1:**
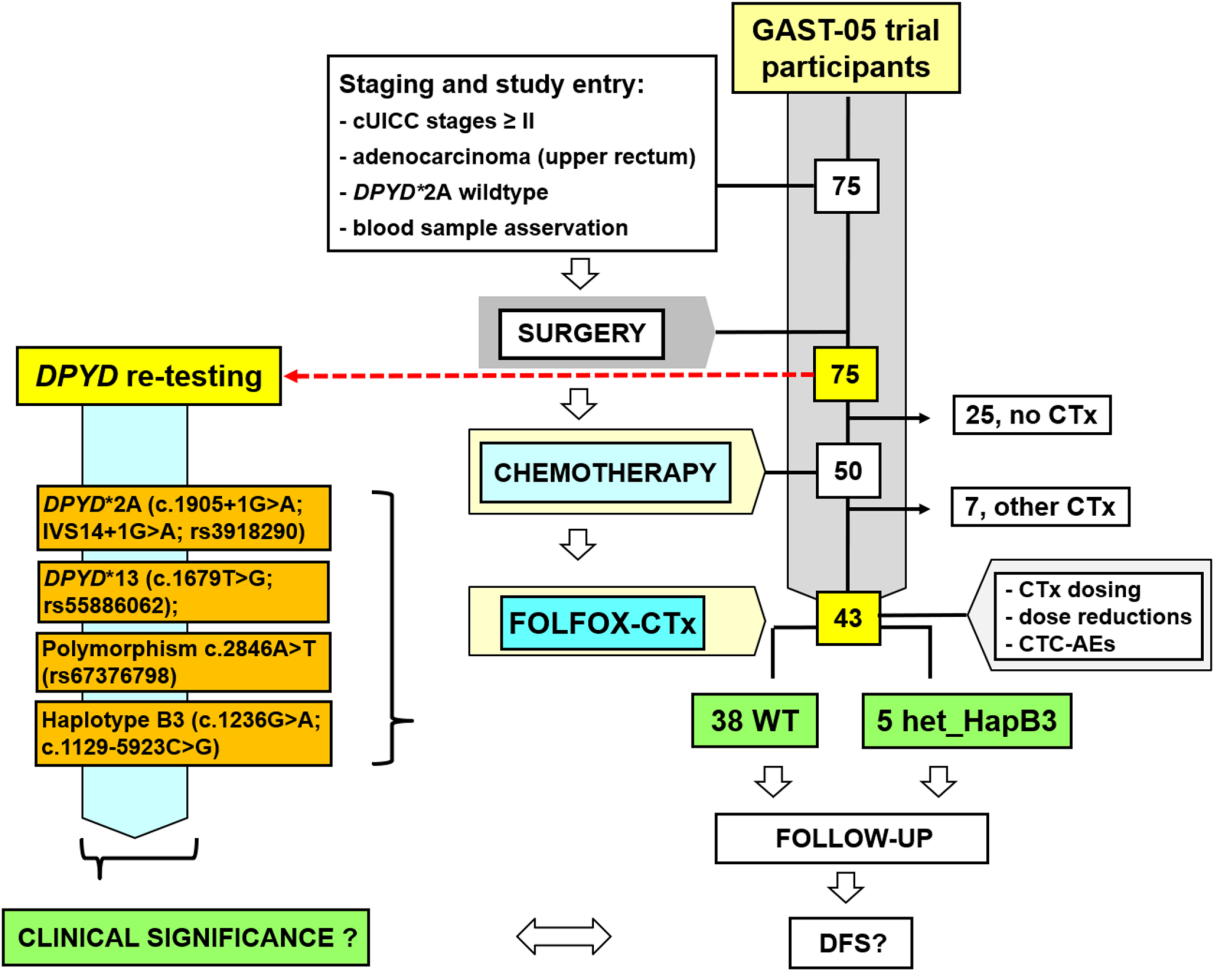
Project design, patients and treatment pathway. After surgery 43 patients with pathologically (p) confirmed adenocarcinomas of pUICC stages II and III (staged according to the current 8th TNM-/UICC-classification [27, 28]) localized in the upper rectum (inferior tumor margin ≥ 12 cm above the anal verge) had been treated with chemotherapy (CTx) according to the GAST-05-trial protocol. FOLFOX-CTx was administered in 38 patients with *DPYD*-wildtype (WT) and in 5 patients with heterozygous Haplotype B3 status (het_HapB3)

A total of 75 patients (median age: 69 years, 26 females; 49 males; Table 1) with locally advanced adenocarcinoma of the upper rectum (inferior tumor margin ≥ 12 cm above the anal verge), clinically staged as cT3/cT4 ± cN0/cN+ (corresponding to UICC stage II or III according to the 8th TNM/UICC-classification [28, 29]), were tested for the DPYD*2A-wildtype (WT) genotype at study entry (Figure A.1). After highly standardized and quality-controlled surgery, FOLFOX-CTx (4 cycles; folinic acid (FA): 400 mg/m², 5-FU: 2.400 mg/m², OX: 100 mg/m²) was started in 43 patients with pathologically confirmed stages II and III (Fig. 1). All patients were treated at the University Medical Center of Göttingen (UMG), Germany, between 03/2007 and 09/2012. The ethics committee at the UMG approved the GAST-05-trial (No 9/8/08) and all patients gave written informed consent. The study was designed and implemented in accordance with the guidelines of Good Clinical Practice (ICH 1996) and the Declaration of Helsinki.

**Table 1:** Characteristics of patients

GAST-05 patients (DPYD*2A-WT)(single study site; *as-treated* data*)*		TME*		PME**		Σ	
		n	%	n	%	n	%
**(n)**	Σ	34	100.0	41	100.0	75	100.0
Patients` sex	Female	9	26.5	17	41.5	26	34.7
	Male	25	73.5	24	58.5	49	65.3
Age (in years)	Mean ± SD	67.14 ± 9.6		67.31 ± 11.97		67.24 ± 10.89	
	Median	68.5		71		69	
	Min-Max	46–87		38–86		38–87	
Age groups (in years)	< 50–59	7	20.6	12	29.3	19	25.3
	60–69	13	38.2	7	17.0	20	26.7
	70 - > 80	14	41.2	22	53.7	36	48.0
Residual status ^1)^	R0	32	94.1	40	97.6	72	96.0
	R1	2	5.9	1	2.4	3	4.0
Circumferential resection status^2)^	≥ 2 mm	23	67.6	22	53.7	45	60.0
	< 2 mm	11	32.4	19	46.3	30	40.0
Tumor (T) status	pT1	1	2.9	1	2.4	2	2.7
	pT2	9	26.5	8	19.5	17	22.7
	pT3	20	58.8	28	68.3	48	64.0
	pT4	4	11.8	4	9.8	8	10.7
Nodal (N) status	pN0	20	58.8	23	56.1	43	57.3
	pN1	6	17.7	12	29.3	18	24.0
	pN2	8	23.5	6	14.6	14	18.7
Distant metastases	pM0	31	91.2	39	95.1	70	93.3
	pM1	3	8.8	2	4.9	5	6.7
L-/V-/Pn- Status ^3)^	Negative	21	61.8	28	68.3	49	65.3
	Positive	13	38.2	13	31.7	26	34.7
Grading of cancer cells	G1	1	2.9	0	0.0	1	1.3
	G2	24	70.6	30	73.2	54	72.0
	G3	9	26.5	11	26.8	20	26.7
UICC-stages*** after surgery	pUICC I	8	23.5	4	9.8	12	16.0
	pUICC II	12	35.3	19	46.3	31	41.3
	pUICC III	11	32.4	16	39.0	27	36.0
	pUICC IV	3	8.8	2	4.9	5	6.7

^(1)^: locoregional residual (R) status after surgery; ^(2)^: circumferential resection margin (CRM) without microscopically detected cancer cells as assessed by the pathologist (p); ^(3)^: peritumoral lymphangiosis carcinomatosa (L1), vascular infiltration (V1) or perineural sheath infiltration (Pn1) by cancer cells; the microscopic detection of any cancer cell infiltration in one of these parameters was sufficient for the diagnosis of pL/V/L-positivity; *TME: total mesorectal excision; **PME: partial mesorectal excision; ***UICC: Union for International Cancer Control [27, 28]; all surgical procedures were performed according to the GAST-05-trial protocol (ISRCTN35198481) and had no influence on the analyses and presented data of this project

Acute toxicity was documented according to the National Cancer Institute Common Terminology Criteria for Adverse Events (CTC-AE), version 3.0 [16]. All CTx dose adjustments were defined in the GAST-05 study protocol. After completion of CTx, patients were followed closely for at least eight weeks and beyond in the case of persistent acute toxicity. For all GAST-05 participants, the follow-up started three months after rectal cancer surgery for a minimum of five years according to the study protocol (the follow-up care plan is summarized in the legend of Figure A.1).

Patients` outcome was assessed using disease-free survival (DFS), defined as the interval from surgery to the first occurrence of local and/or distant recurrence, secondary malignancy, or death from any cause. Patients with lost to follow-up were censored at the date of last contact.

For molecular biological analyses, cryopreserved blood samples were used for extended *DPYD* testing. Genomic DNA was isolated using the QIAamp^®^ DNA Blood Minikit from QIAGEN (Hilden, Germany) following the manufacturer`s instructions. To detect the five *DPYD-*variants (Table A.2) polymerase chain reaction (PCR) using a melting curve approach was carried out on a LightCycler^®^ 480 II (Roche Diagnostics, Mannheim, Germany) quantitative real-time-PCR-system (Table A.3).

### Statistics

The maximum toxicity (per FOLFOX cycle) was summarized using absolute and relative frequencies and compared between patients with WT vs. *DPYD-*mutations using Fisher’s exact test. Total CTC-AEs were summarized by group, domain and grade. Mixed logistic regression models were fit to study the effect of *DPYD*-mutations on CTC-AEs (high/low grade) including a random intercept per patient to account for repeated measures. Similar models were fit for the endpoint dose reduction. Additional co-variables were included as indicated in the tables and text. Postoperative DFS was calculated using the Kaplan-Meier estimator, and survival curves were compared using the log-rank test. Additionally, the effects of *DPYD*-mutations and the relative administered 5-FU dose onto DFS were tested using a proportional hazard Cox-regression model adjusting for age and sex. The resulting model coefficients are reported as hazard ratios (HRs) with 95% confidence intervals (95%-CI) and p-values testing the null-hypothesis of no association. The significance level was set to alpha = 5%. All analyses were done with the statistics software R (version 4.2.3; [29]) using the R-package lme4 (version 1.1.32; [30]) for the mixed effect logistic regression.

## Results

*DPYD* re-testing revealed 100% WT for DPYD*2A, DPYD*13 and the polymorphism c.2846 A > T. Interestingly, in five patients a het_HapB3 status was newly diagnosed post-hoc (Table A.4). Patients` characteristics are summarized in Table 1. In total, 43 patients with histopathological confirmed LARC stages II or III had started the FOLFOX-CTx (Fig. 1).

The adherence to CTx cycle 1 to 4 was 100.0%, 97.7%, 95.3% and 93.0%, respectively. For the 38 patients with *DPYD*-WT, the administered dosages of 5-FU and OX were 92.6% and 75.3%, and for the 5 het_HapB3-patients 68.8% and 55.0% of the target dose, respectively.

**Fig. 2:**
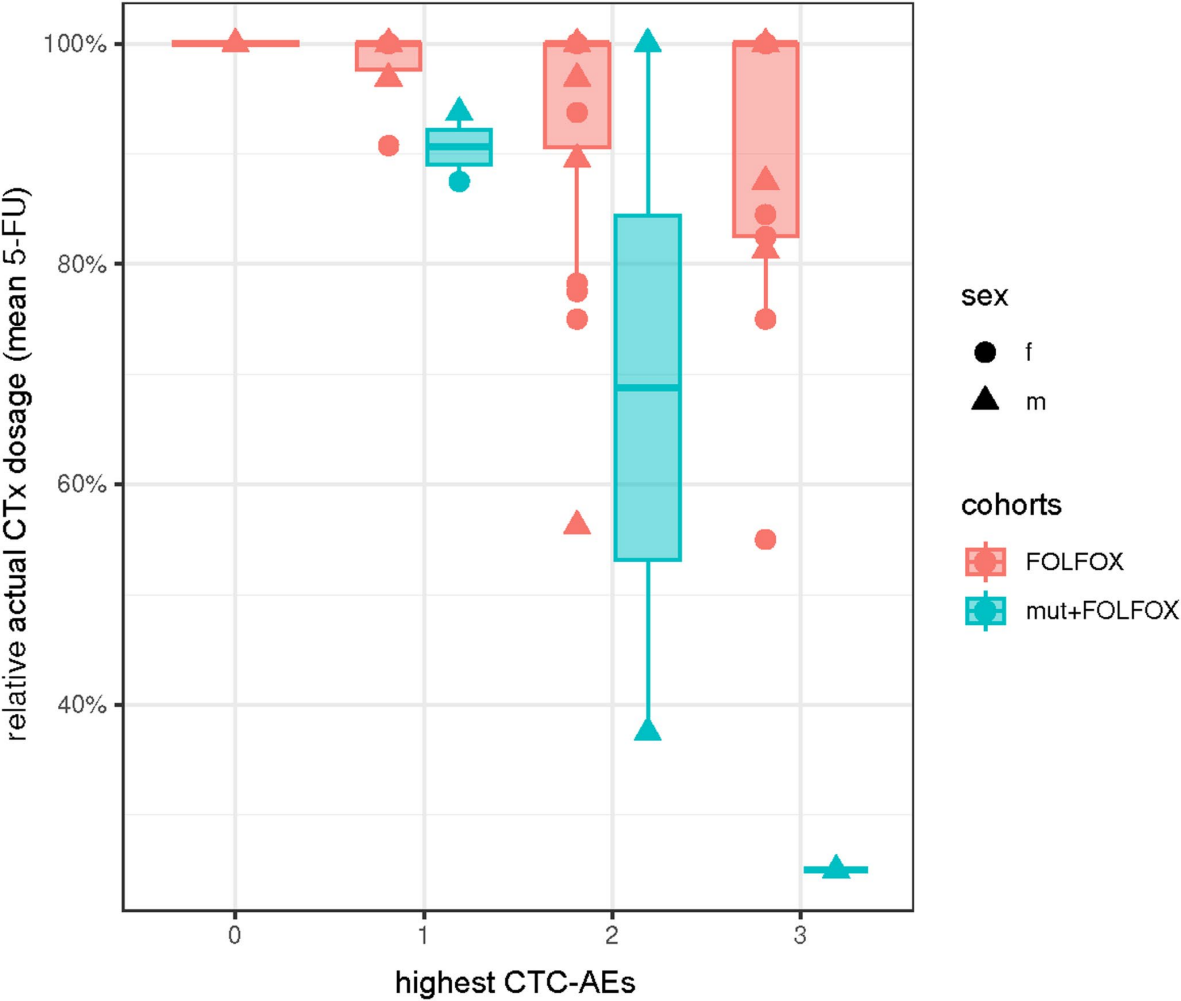
Lower 5-FU doses for patients with het_***DPYD*** -Haplotype B3. The 5-FU dosages over all FOLFOX-cycles are shown in relation to the highest CTC-AE grades (NCI-Common Toxicity Criteria of Adverse Events, version 3.0 [16]) and to the *DPYD*-status of all 43 patients. The patients were grouped in two cohorts according to their *DPYD*-wildtype (WT, red) or *DPYD*-mutational status (mut, blue) after post-hoc genotyping for the most common *DPYD*-variants. For visualization box plots overlayed with single points were used; f: female; m, male

The cumulative CTx dose relative to the planned CTx dose across all cycles, grouped by the highest observed CTC-AE grade and *DPYD* mutation, is shown in Fig. 2. Compared to the WT patients, the five het_HapB3 patients had received substantially lower 5-FU doses. The number of CTC-AEs in all five het_HapB3 patients are visualized independently of the CTC-AE severity per CTx cycle. Adjustments of the 5-FU dose (expressed as the relative actual 5-FU dose) were implemented in accordance to standardized procedures defined in the GAST-05 trial protocol based on the highest observed CTC-AE grade.

Figure A.2 illustrates the impact of these 5-FU dose modifications in the het_HapB3 carriers under FOLFOX-CTx. In two patients (01_mutCTx, 03_mutCTx), a relatively high incidence of CTx-associated AEs led not only to a reduction in the 5-FU dose, but ultimately to discontinuation of CTx. In contrast, the treatment course of patient 02_mutCTx demonstrates that a lower rate of CTC-AEs during the first two CTx cycles enabled administration of the planned 5-FU dose.

The maximum toxicity per CTx cycle was summarized based on absolute and relative CTC-AE frequencies and compared between patients ± *DPYD*-mutation (Table A.5). In this patient-level analysis, there was no significant association between mutation status and the highest grade of toxicity. Furthermore, the total number of CTC-AEs (all grades) was calculated by patients` group and CTC-AE grade (Table A.6). We observed comparable percentages of low grade (G1-G2, 24.7% and 26.6%) and high grade (G ≥ 3, 0.9% and 0.9%) CTx-associated toxicity in the *DPYD*-WT and het_HapB3 groups. In addition, CTx-associated toxicity was assessed in 14 areas (Table A.7). Detailed information on CTC-AEs per area, differences per area during the first CTx cycle and between patient groups (WT vs. het_HapB3 patients) is given in Tables A.8 to A.10.

Mixed logistic regression models were fitted to examine the mutational effect on the grade (high vs. low) of toxicity events including a random intercept per patient to account for the repeated measures. In a first model, only toxicity events at the 1 st cycle of FOLFOX were analyzed excluding four patients, that had received < 100% of the planned 5-FU dose. No significant differences between *DPYD-*WT- and het_HapB3 patients were observed [OR: 3.00; 95%-CI: 0.03–346.87; *p* = 0.650] (Table 2A).

**Table 2:** CTx-associated high grade toxicity in patients´ group

	Risk for high-grade CTC-AEs (G ≥3) under CTx			
	Predictors	OR	CI	p-value
A:CTx cycle 1	(Intercept)	0.00	0.00 – 0.04	**0.001**
	Group [het_HapB3+FOLFOX]	3.00	0.03 – 346.87	0.650
	N _study_patient number_	39		
	Observations	554		
	Marginal R^2^ / conditional R^2^	0.006 / 0.861		
B: CTx cycles 2-4	(Intercept)	0.10	0.00 – 0.02	**<0.001**
	3^rd^ CTx cycle	1.47	0.48 – 4.54	0.502
	4^th^ CTx cycle	0.64	0.16 – 2.50	0.521
	Group [het_HapB3+FOLFOX]	0.18	0.01 – 3.73	0.270
	Δ relative current 5-FU dosage	5.51	0.33 – 93.02	0.236
	N _study_patient number_	42		
	Observations	1755		
	Marginal R^2^ / conditional R^2^	0.089 / 0.110		

The table shows regression coefficients from a mixed effect logistic regression model for high-grade toxicity on CTC-AEs assessed in 14 areas. A: No significantly increased risk for high-grade CTC-AEs (G≥3) was found in patients with het_*DPYD*-Haplotype B3 (het_HapB3) compared to *DPYD*-WT during the first CTx cycle. B: No significant difference between *DPYD*-WT- and het_HapB3-patients was observed (OR: 0.18; 95 %-CI: 0.01-3.73; p = 0.270). CTx: chemotherapy; CTC-AE: NCI-Common Toxicity Criteria of Adverse Events, version 3.0 [16]; OR: odds ratio; 95 %-CI: 95% confidence interval

In a second model the grade (high vs. low) of CTC-AEs in CTx cycles 2–4 was analyzed with the mutational status as a predictor, additionally controlling for cycle and dose reduction from the previous cycle (Table 2B). No significant difference between *DPYD*-WT- and het_HapB3-patients was observed [OR: 0.18; 95%-CI: 0.01–3.73; *p* = 0.270].

Furthermore, there was no significantly higher risk for any toxicity at the first FOLFOX cycle for het_HapB3 carriers, when all grades of CTC-AEs were considered [OR: 1.84; 95%-CI: 0.77–4.37; *p* = 0.170] (Table A.11).

Compared to CTx cycle 2, there was a higher chance for patients to suffer AEs at later cycles: [at 3rd CTx cycle - OR: 1.28; 95%-CI: 0.97–1.68; *p* = 0.076; at 4th cycle - OR: 1.33; 95%-CI: 1.02–1.75; *p* = 0.038] (Table A.12). The dose reduction from previous FOLFOX cycle shows a strong impact on the occurrence of CTC-AEs during treatment [OR: 2.67; 95%-CI: 1.00–7.12; *p* = 0.051]. Again, no significant difference was found between patients with *DPYD*-WT- and het_HapB3-status [OR: 0.93; 95%-CI: 0.41–2.13; *p* = 0.873].

In total, 26 (60.5%) GAST-05 participants of our study site had received full dose of FOLFOX (Table A.13). Logistic regression analysis revealed the het_HapB3-status (*p* = 0.044) and male sex (*p* = 0.049) as predictors for 5-FU dose-reductions during postsurgical FOLFOX-CTx (Table A.14).

At the CTx cycle level, the prediction of dose reduction narrowly failed to reach statistical significance for the mutation cohort (*p* = 0.053 Table A.15). In addition, when looking at the number of 5-FU dose reductions at the patient level, study participants with newly diagnosed het_HapB3 status had a higher, but narrowly non-significant risk of 5-FU dose reductions (*p* = 0.06, Table A.16) below the cut-off of 75% of the planned 5-FU dose compared to *DPYD*-WT carriers. It was then hypothesized that both the *DPYD* mutation status and any 5-FU dose reduction may have had an effect on DFS.

**Fig. 3:**
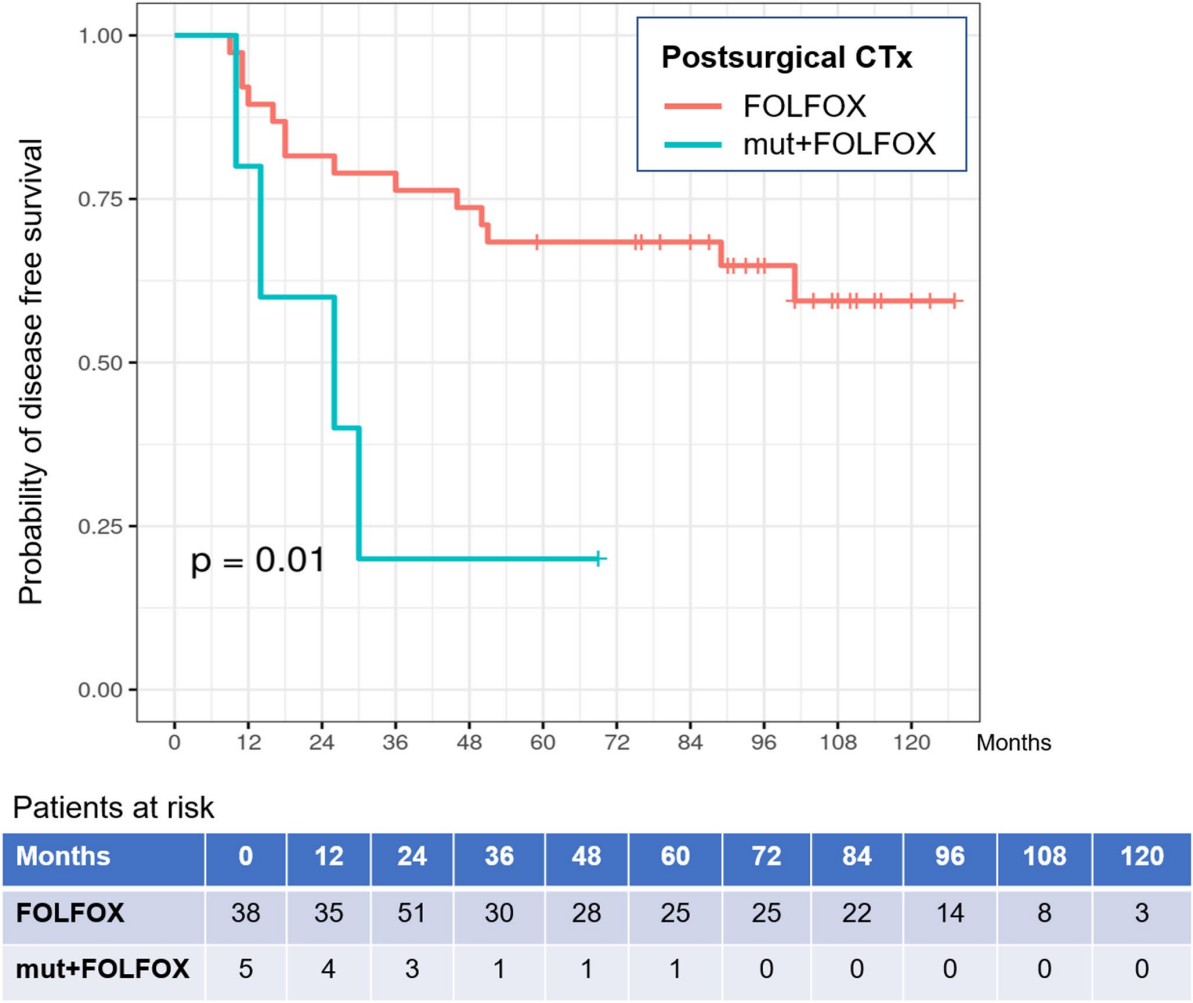
DFS in patients with ***DPYD***-WT- and het_***DPYD***-Haplotype B3-status. The Kaplan-Meier estimator demonstrates the disease-free survival (DFS) probability for 38 *DPYD-*WT- patients vs. 5 het_Haplotype B3-patients (mut). All these patients had started postsurgical treatment with FOLFOX chemotherapy (CTx); logrank test: *p* = 0.01

During long-term follow-up (median: 101 months), events for DFS were detected in 14 (36.8%) WT-patients and in four (80.0%) het_HapB3 carriers. Patients with this newly diagnosed mutational status demonstrated a significantly reduced DFS probability as compared to WT study participants (logrank test: *p* = 0.010) (Fig. 3).

In the multivariable Cox regression model for DFS (Table A.17), the presence of a *DPYD* mutation was associated with a higher risk of reduced DFS [HR: 3.774; 95%-CI: 0.96–14.8; *p* = 0.057].

Interestingly, 5-FU dose reductions (of any amount) did not show significance in DFS between GAST-05 patients with and without 5-FU dose reductions (Figure A.3).

## Discussion

This retrospective analysis evaluated the clinical impact of previously undetected *DPYD* mutations, at the time of treatment, in patients undergoing 5-FU-based CTx for upper rectal cancer. Our findings raise critical considerations for personalized oncology.

Contrary to expectations, we did not observe a significant association between het_HapB3 status and CTx-associated toxicity [25, 30–32]. Across multiple assessments, including the number and grade of AEs, cycle-specific toxicity, and mixed logistic regression models controlling for repeated measures and covariates, het_HapB3 mutation carriers experienced toxicity rates comparable to WT patients. In both groups no significant difference in any-grade toxicity was observed during the first FOLFOX cycle (OR 1.84; *p* = 0.170), and subsequent cycles.

Despite these similar toxicity profiles, het_HapB3 carriers experienced significantly reduced DFS, with events recorded in 80% of mutation carriers versus 36.8% of WT patients (log-rank *p* = 0.010). Multivariable Cox regression analysis identified HapB3 status as a near-significant predictor of reduced DFS (HR 3.77; *p* = 0.057), suggesting a potential prognostic role independent of overt toxicity or dose adjustments.

Moreover, logistic regression revealed that het_HapB3 status was a predictor of 5-FU dose reductions during postoperative therapy (*p* = 0.044), with a strong association at the patient level (*p* = 0.06). Interestingly, 5-FU dose reductions were not significantly associated with DFS in the total population (Figure A.3), suggesting that the impaired DFS observed in het_HapB3 carriers may be driven by intrinsic metabolic or tumor-associated pathways.

However, as no 5-FU plasma level assessments and subsequent 5-FU therapeutic drug monitoring (TDM) were performed and only the four most common *DPYD*-variants were analysed post-hoc, final conclusions cannot be drawn. Due to the missing TDM in this study, only administered doses were available and we cannot rule out that there might be an effect of 5-FU plasma levels onto DFS. Also, the data are observational with respect to the 5-FU dose administered, and the clinically indicated dose adjustments might work well enough to shadow long term effects in a cohort of this size. One hypothesis regarding the impact of *DPYD* mutations is that reduced DPD enzyme activity resulted in elevated plasma 5-FU concentrations, which may not have translated into increased antitumor efficacy. This pharmacokinetic imbalance might explain why DFS was not significantly associated with 5-FU dose reductions. From the clinical point of view this issue is particularly relevant in LARC, where the therapeutic window is narrow and local tumor control is critical.

At the beginning of the GAST-05 trial, no general recommendations for *DPYD* genotyping existed. The decision to implement DPYD*2A testing—though innovative and, at the time, subject to debate among some study groups and oncologists—was made at the discretion of the investigators. According to the current guidelines of the EMA and DGHO, het_HapB3 carriers would have received reduced initial doses of 5-FU (Table 3) [25, 32, 34, 35]. This raises another important concern: could genotype-based dose reductions, while minimizing the feared toxicity, lead to a functional underdose and compromised therapeutic efficacy in mutation carriers? Our findings support the argument that genotyping alone may not be sufficient to guide MMT for LARC patients. Measuring 5-FU plasma levels at defined timepoints followed by 5-FU TDM seems to be mandatory to personalize dosing precisely, safe and most effectively [36–38].

**Table 3:** *DPYD*-variants, DPD enzyme activity and recommended 5-FU dosing

Genotype	Sequence variant	dbSNP (rs#)	Allele	DPD-enzyme		5-FU dose (in %) ^1)^
				Activity	Score	
DPYD*1	Wildtype	–	Homo	Normal	2	100%
DPYD*2A	c.1905 + 1G > A	rs3918290	Homo	None	0	no 5-FU
			Hetero	▼▼	1	50%
DPYD*13	c.1679T > G	rs55886052	Homo	None	0	no 5-FU
			Hetero	▼▼	1	50%
Polymorphism	c.2846 A > T	rs67376798	Homo	▼▼▼	0.5 ^**2) 3)**^	25 - 50%^**2)**^
			Hetero	▼	1.5	50 - 75% ^**2) 3)**^
Haplotype B3	c.1236G > A	rs56038477	Homo	▼▼▼	0.5 ^**3)**^	25 - 50%^**2)**^
			Hetero	▼	1.5	50 - 75% ^**2) 3)**^
	c.1129–5923 C > G	rs75017182	Homo	▼▼▼	0.5 ^**3)**^	25 - 50%^**2)**^
			Hetero	▼	1.5	50–75% ^**2)**^

^**1)**^: adapted 5-FU dosage (in % of the planned 5-FU dose); ^**2)**^: additional phenotyping (as measured by plasma uracil ± dihydrouracil concentrations, or by enzyme activity assessment in peripheral blood mononuclear cells, PBMC) for determination of patient`s DPD enzyme activity is recommended [38]; ^**3)**^: there is a difference considering the DPD enzyme activity score of 1 vs. 0.5 as recommended in the guidelines of the CPIC (Clinical Pharmacogenetics Implementation Consortium [32] and the position paper of the German Society of Haematology and Oncology (DGHO) [36]); hetero: heterozygous; homo: homozygous; dbSNP (rs#): database of single nucleotide polymorphism (SNP)

Taken together, this study provides the first evidence, to our knowledge, that HapB3 is associated with impaired DFS in patients with LARC in the upper rectum treated with 5-FU-based adjuvant CTx, even in the absence of increased toxicity. These findings support the inclusion of HapB3 in *DPYD* screening panels and highlight the limitations of relying solely on toxicity or single genotypes to guide treatment. Prospective clinical trials integrating pharmacogenetics, drug-level monitoring, and oncological outcome parameters are mandatory to develop a more comprehensive framework for an individualized 5-FU-based (multimodal) treatment.

Notably, HapB3 prevalence was 11.6% in our cohort, slightly higher than previously reported frequencies (2.6–6.3%) among Caucasians [34, 39]. All identified carriers presented with the full HapB3 haplotype; no incomplete genotypes were observed. The linkage of such variants has been questioned [40]. Recently, our group has reported a previously unknown constellation of an incomplete HapB3 genotype, presenting only the synonymous c.1236G > A without c.1129–5923 C > G variant [41].

Although HapB3 has been associated with variable DPD enzyme activity (ranging from near-normal to severely reduced), the functional significance of synonymous variants such as c.1236G > A remains debated [36]. Previous studies report up to 40% reduction in DPD activity in heterozygous carriers, and up to 70% in homozygous individuals, although functional retention has also been described [31, 33, 34, 36, 42, 43]. This heterogeneity underscores the need for individualized pharmacokinetic assessment, too.

Our findings are in line with reports from the ALPE study, which showed reduced progression-free survival in HapB3 carriers receiving dose-reduced fluoropyrimidines [44]. Likewise, the FUSAFE meta-analysis questioned the utility of a 25–50% dose reduction in heterozygous carriers given the relatively low risk of severe (grade ≥ 4) toxicity [45]. These findings are further supported by data on genotype-phenotype discrepancies, incomplete haplotypes, and the existence of rare *DPYD*-variants not covered by standard panels - all reinforcing the role of TDM in modern 5-FU based MMT [46, 47].

Although our study addresses relevant clinical questions, it has some limitations. First, we only present monocentric data with a small number of patients. We are well aware, that our findings should be subject to validation in larger LARC populations from multicenter trials. In addition, analytical techniques that test the dihydrouracil/uracil ratio in blood plasma or the individual DPD activity in peripheral blood mononuclear cells may increase the effectiveness of DPD screening [25, 36]. These procedures are not yet standard in many countries but could improve the results of *DPYD* genotyping [36]. Finally, routine assessment of 5-FU plasma levels followed by 5-FU dose escalation was not performed, but should be considered in future innovative trials using 5-FU with much higher safety and efficacy.

## Conclusion

This retrospective analysis highlights the clinical relevance of *DPYD* pharmacogenetics in rectal cancer patients undergoing 5-FU-based chemotherapy. Although carriers of the het_HapB3-variant did not exhibit significantly increased treatment-related toxicity, they demonstrated a markedly reduced DFS. This discrepancy suggests that *DPYD*-variants may influence not only toxicity profiles but also therapeutic efficacy, independently of overt adverse events or dose modifications.

Our findings support current recommendations for pre-treatment *DPYD* genotyping and further suggest that genotype-guided dose adjustments alone may be insufficient to ensure both treatment safety and efficacy. The observation of impaired DFS despite similar toxicity and dosing underscores the likelihood that 5-FU exposure levels in mutation carriers are often suboptimal. Therefore, integrating genetic screening with TDM could provide a more robust and individualized approach to fluoropyrimidine dosing. Prospective studies incorporating pharmacokinetic profiling and functional enzyme activity assessments are mandatory to validate this approach and to improve treatment outcomes, particularly for carriers of reduced-function *DPYD*-variants, such as HapB3.

## Electronic supplementary material

Below is the link to the electronic supplementary material.

Supplementary Material 1

## Data Availability

No datasets were generated or analysed during the current study.
