# Supplementary material for: Unrecognized mutations in DPYD* 2 A wild-type rectal cancer patients receiving postoperative 5-FU-based chemotherapy - do they have a clinical impact?
Source: Cancer Chemother Pharmacol. 2025 Jul 15;95(1):75. doi: 10.1007/s00280-025-04787-4 (PMC12259744; doi:10.1007/s00280-025-04787-4)
Supplement: Supplementary file 1 — Supplementary Material 1 [file 280_2025_4787_MOESM1_ESM.docx]

**Appendix: Supplemental material**

| ***DPYD* mutational variants** | **Toxicity grade ≥ 3** (5-FU civ or bolus) (OR / *RR [95 %-CI], p-value) | **Literature** (studies, patients) |
| --- | --- | --- |
| *DPYD**2A (rs3918290) | overall toxicity (OR: 5.42; 95 %-CI:2.79 – 10.52; p < 0.001) | Terrazzino et al. 2013 ^1)^ 15 studies (n = 6.402) |
| c.2846A>T (rs67376798) | overall toxicity (OR: 8.18; 95 %-CT:2.65 – 25.25; p < 0.001) |  |
| *DPYD**2A (rs3918290) | overall toxicity (OR: 6.71; 95 %-CI: 1,66 – 27.21; p = 0.0075) | Rosmarin et al. 2014 ^2)^  17 studies (n = 4.855) |
| *DPYD**2A (rs3918290) | overall toxicity (RR: 2.85; 95 %-CI: 1.75 – 4.62; p < 0.0001) | Meulendijks et al. 2015 ^3)^ 8 studies (n = 7.365) |
| c.2846A>T (rs67376798) | overall toxicity (RR: 3.02; 95 %-CI: 2.22 – 4.10; p < 0.0001) |  |
| *DPYD**13 (c.1679T>G; rs55886062) | overall toxicity (RR: 4.40; 95 %-CI: 2.08 – 9.30]; p < 0.0001) |  |
| Haplotype B3 (c.1236G>A; (rs56038477) | overall toxicity (RR: 1.59; 95 %-CI: 1.29 – 1.97]; p < 0.0001) |  |

**Table A.1: Toxicity associations of *DPYD* variants in meta-analysis**

^1)^ Terrazzino et al. had analyzed 4.094 patients (from 15 studies) for *DPYD**2A (IVS14+1G>A; rs3918290) and 2.308 patients for c.2846A>T (D949V, rs67376798) pointing out the clinical validity of these mutations as risk factors for fluoropyrimidine-associated severe toxicities [24]. ^2)^ Rosmarin et al. had assessed data of 4.855 patients (from 17 studies) by testing for 8 *DPYD*-variants. Carriers with the *DPYD**2A and c.2846A>T variants had also suffered severe toxicities, when exposed to 5-FU [25]. ^3)^ The meta-analysis by Meulendijks et al. included data from 7.365 patients (from 8 studies) and confirmed the association between high-grade CTC-AEs and the *DPYD**2A or c.2846A>T variants, but also for the *DPYD**13; c.1679T>G; rs55886062) and c.1236G>A / Haplotype B3 (rs56038477) variants, respectively [26]; *RR: relative risk; OR: odds ratio; CI: 95% confidence interval; modified table according to [27]; CTC-AE: NCI-Common Toxicity Criteria of Adverse Events, version 3.0 [17].

| **Primer name** | **Primer sequence 5'-->3'** |
| --- | --- |
| DPD_Ex14for | 5'-ATGTATGGCCCTGGACAAAG-3' |
| DPD_Ex14rev | 5'-ATGCATCAGCAAAGCAACTG-3' |
| DPD_Ex14anchor3+ | 5'-Cy5-TAACATCTAAAACAAGAGAATTGGCATAAGTTGG-Pho-3' |
| DPD_Ex14wt-probe3+ | 5'-AGACAACGTAAGTGTGAT–Flu-3' |
| DPD_2846for | 5'-AGCACTGCAGTACCTTGGAA-3' |
| DPD_2846rev | 5'-TCTAATTCCAGCAGGATTCTTACC-3' |
| DPD_2846anchor+ | 5'-Cy5-TCAACTGTGGTAAATGCTACATGACCTGTAAT-Pho-3' |
| DPD_2846mut-probe+ | 5'-TGGCTATGATTGTTGAAGAAATGT-Flu-3' |
| DPYD_*13 for | 5'-AAGTTTTGGTGAGGGCAAAA-3' |
| DPYD_*13rev | 5'-CAAGCCTGAACTACCCCTCTT-3' |
| DPYD_*13anchor | 5'-CY5-GGCTGGAGTTGCGCTAGCAAGACC-PHO-3' |
| DPYD_*13mut_probe | 5'-PHO-CTTCGACTCATTGATGTGCT-FLU -3' |
| DPYD_HapB3_1236for | 5'-TTTCTGCCATTCCTGTCCCC-3' |
| DPYD_HapB3_1236rev | 5'-CAGAACTGAACCAAAGGCAC-3' |
| DPYD_HapB3_1236anchor | 5'-PHO-AGGTTATAGTAAAAAGGTGGGAGAATTGTTGCTATG-FLU-3' |
| DPYD_HapB3_1236wt-probe | 5'-Cy5-TTTGTTCGGACAGAGCAAGA-PHO-3' |
| DPYD_HapB3_1129for | 5'-TCACTCAGCATCAGCCACAT-3' |
| DPYD_HapB3_1129rev | 5'-AAGCAAACATGCCAACCTCT-3' |
| DPYD_HapB3_1129anchor | 5'-Cy5-ATTTTTTTTAACTTTCCCACTACTGTGTAAAAAGAAAGTG-PHO-3' |
| DPYD_HapB3_1129wt-probe | 5'-PHO-ATGGAGGTGAAAATCAAAGCTG-FLU-3' |

**Table A.2: Primer and probe sequences**

A sequence-specific primer set and wildtype (WT)- or mutation-specific probes were designed for each of the five *DPYD-*variants.

| **A** | **°C** | **Seconds** | **Cycles** |  | **B** | **Final Concentration** |
| --- | --- | --- | --- | --- | --- | --- |
| initial denaturation | 95 | 120 | 1 |  | 10x KCl buffer (15 mM) | 1x 1,5mM |
| denaturation | 95 | 10 | x45 |  | MgCl_2_ (50 mM) | 1 mM |
| annealing | 55 | 10 |  |  | DMSO | 5% |
| extension | 72 | 20 |  |  | dNTP’s (10mM) | 0,2 mM |
| melting | 95 | 30 |  |  | DNA polymerase 5 U/µL | 1 U |
|  | 30 | 30 |  |  | primerMix | 0,1 - 1µM |
|  | 85 |  | acquisition |  | genomic DNA | 1µl |
|  | 37 | ꚙ |  |  | ddH2O | add to 20µl |

**Table A.3: PCR conditions**

All *DPYD* genotyping was done according to well-established methods and blinded to clinical data. A: PCR cycling program used for amplification; B: final concentrations of PCR reagents used.

| **Patient study ID** | ***DPYD*-variants** | | | | | |
| --- | --- | --- | --- | --- | --- | --- |
|  | ***DPYD**2A** | | ***DPYD**13** | **Polymorphism (c.2846A>T)** | **Haplotype B3  (c.1129-5923C>G)** | **Haplotype B3 (c.1236G>A)** |
|  | **initially** | **actually** |  |  |  |  |
| 81001 | WT | WT | WT | WT | WT | WT |
| 81003 | WT | WT | WT | WT | WT | WT |
| 81004 | WT | WT | WT | WT | WT | WT |
| 81005 | WT | WT | WT | WT | WT | WT |
| 81007 | WT | WT | WT | WT | WT | WT |
| 81010 | WT | WT | WT | WT | WT | WT |
| 81014 | WT | WT | WT | WT | WT | WT |
| 81016 | WT | WT | WT | WT | WT | WT |
| 81017 | WT | WT | WT | WT | WT | WT |
| 81018 | WT | WT | WT | WT | WT | WT |
| 81019 | WT | WT | WT | WT | WT | WT |
| 81021 | WT | WT | WT | WT | WT | WT |
| 81022 | WT | WT | WT | WT | WT | WT |
| 81024 | WT | WT | WT | WT | WT | WT |
| 81025 | WT | WT | WT | WT | WT | WT |
| 81026 | WT | WT | WT | WT | WT | WT |
| 81027 | WT | WT | WT | WT | WT | WT |
| 81028 | WT | WT | WT | WT | WT | WT |
| 81029 | WT | WT | WT | WT | WT | WT |
| 81030 | WT | WT | WT | WT | WT | WT |
| 81032 | WT | WT | WT | WT | het | het |
| 81033 | WT | WT | WT | WT | WT | WT |
| 81034 | WT | WT | WT | WT | WT | WT |
| 81035 | WT | WT | WT | WT | WT | WT |
| 81036 | WT | WT | WT | WT | WT | WT |
| 81037 | WT | WT | WT | WT | WT | WT |
| 81038 | WT | WT | WT | WT | WT | WT |
| 81043 | WT | WT | WT | WT | WT | WT |
| 81045 | WT | WT | WT | WT | WT | WT |
| 81046 | WT | WT | WT | WT | WT | WT |
| 81047 | WT | WT | WT | WT | WT | WT |
| 81048 | WT | WT | WT | WT | WT | WT |
| 81049 | WT | WT | WT | WT | WT | WT |
| 81050 | WT | WT | WT | WT | WT | WT |
| 81051 | WT | WT | WT | WT | WT | WT |
| 81052 | WT | WT | WT | WT | WT | WT |
| 81053 | WT | WT | WT | WT | WT | WT |
| 81054 | WT | WT | WT | WT | WT | WT |
| 81056 | WT | WT | WT | WT | WT | WT |
| 81057 | WT | WT | WT | WT | WT | WT |
| 81058 | WT | WT | WT | WT | het | het |

(continued from table A.4)

| 81059 | WT | WT | WT | WT | WT | WT |
| --- | --- | --- | --- | --- | --- | --- |
| 81060 | WT | WT | WT | WT | WT | WT |
| 81061 | WT | WT | WT | WT | het | het |
| 81064 | WT | WT | WT | WT | WT | WT |
| 81065 | WT | WT | WT | WT | WT | WT |
| 81066 | WT | WT | WT | WT | WT | WT |
| 81067 | WT | WT | WT | WT | WT | WT |
| 81069 | WT | WT | WT | WT | WT | WT |
| 81070 | WT | WT | WT | WT | WT | WT |
| 81071 | WT | WT | WT | WT | WT | WT |
| 81072 | WT | WT | WT | WT | WT | WT |
| 81073 | WT | WT | WT | WT | WT | WT |
| 81074 | WT | WT | WT | WT | WT | WT |
| 81075 | WT | WT | WT | WT | WT | WT |
| 81076 | WT | WT | WT | WT | het | het |
| 81077 | WT | WT | WT | WT | WT | WT |
| 81078 | WT | WT | WT | WT | WT | WT |
| 81079 | WT | WT | WT | WT | WT | WT |
| 81081 | WT | WT | WT | WT | WT | WT |
| 81083 | WT | WT | WT | WT | WT | WT |
| 81084 | WT | WT | WT | WT | WT | WT |
| 81085 | WT | WT | WT | WT | WT | WT |
| 81086 | WT | WT | WT | WT | WT | WT |
| 81088 | WT | WT | WT | WT | WT | WT |
| 81089 | WT | WT | WT | WT | WT | WT |
| 81090 | WT | WT | WT | WT | WT | WT |
| 81091 | WT | WT | WT | WT | WT | WT |
| 81092 | WT | WT | WT | WT | WT | WT |
| 81093 | WT | WT | WT | WT | WT | WT |
| 81095 | WT | WT | WT | WT | WT | WT |
| 81096 | WT | WT | WT | WT | WT | WT |
| 81097 | WT | WT | WT | WT | WT | WT |
| 81098 | WT | WT | WT | WT | WT | WT |
| 81099 | WT | WT | WT | WT | het | het |

**Table A.4: Testing for the most common *DPYD*-mutations**

Patient study ID: patient`s identification number; *DPYD**2A initially: at staging previously before study entry an exon 14 skipping test for detection of *DPYD**2A mutations was performed in all 75 participants of the GAST-05-trial; *DPYD**2A actually: for the current post-hoc study, cryopreserved blood samples of all 75 patients were re-examined (blinded to the previous findings) for the most common *DPYD*-mutations including *DPYD**2A-variants; there was a 100 % matching between the previous and current *DPYD**2A tests. WT: wildtype; het_type: heterozygous type.

| **Parameter** | **level** | **FOLFOX-CTx** | **mut+FOLFOX-CTx** | **p-value ¹^)^** | **adjusted p-value ²^)^** |
| --- | --- | --- | --- | --- | --- |
| **n (patients)** |  | **38** | **5** |  |  |
| **1^rst^ CTx cycle** |  |  |  | 0,40 | 1,0 |
| CTC-AEs (grades ≤ 2) | low | 35 (92,1%) | 4 (80,0%) |  |  |
| CTC-AEs (grades ≥ 3) | high | 3 (7,9%) | 1 (20,0%) |  |  |
| **2^nd^ CTx cycle** |  |  |  | 1,00 | 1,0 |
| CTC-AEs (grades ≤ 2) | low | 36 (94,7%) | 5 (100,0%) |  |  |
| CTC-AEs (grades ≥ 3) | high | 2 (5,3%) | 0 (0,0%) |  |  |
| **3^rd^ CTx cycle** |  |  |  | 1,00 | 1,0 |
| CTC-AEs (grades ≤ 2) | low | 33 (86,8%) | 5 (100,0%) |  |  |
| CTC-AEs (grades ≥ 3) | high | 5 (13,2%) | 0 (0,0%) |  |  |
| **4^th^ CTx cycle** |  |  |  | 1,00 | 1,0 |
| CTC-AEs (grades ≤ 2) | low | 36 (94,7%) | 5 (100,0%) |  |  |
| CTC-AEs (grades ≥ 3) | high | 2 (5,3%) | 0 (0,0%) |  |  |

**Table A.5: CTx- associated toxicity (CTC-AE-grading) per patient**

Separately for each CTx-cycle the association between high-grade CTC-AEs (grades ≤ 2 vs grades ≥ 3) and *DPYD*-mutational status was tested using Fisher`s exact test for counted data. This table shows for each CTx cycle a cross tabulation and the resulting p-values. ¹ p-values from Fisher's exact test; ² adjusted p-values using the Bonferroni-Holm correction; CTC-AE grading was used according to the National-Cancer-Institute Common Terminology Criteria for Adverse Events, version 3.0 [17].

| **Parameter** | **level** | **WT + CTx** | **%** | **mut + CTx** | **%** |
| --- | --- | --- | --- | --- | --- |
| **n (events, all grades)** |  | 2167 | 100.0% | 284 | 100.0% |
| **CTC-AEs grouped** |  |  |  |  |  |
| CTC-AE (G0) | 0 | 1600 | 74.4% | 155 | 72.4% |
| CTC-AEs (G1 - G2) | low | 532 | 24.7% | 57 | 26.6% |
| CTC-AEs (G ≥3) | high | 19 | 0.9% | 2 | 0.9% |
| (missing) |  | 16 |  | 70 |  |

**Table A6: CTx-associated toxicity in patients´ group**

Comparable percentages of low grade (G1-G2) and high grade (G≥3) CTx-associated toxicity (CTC-AEs) were found in GAST-05 participants with *DPYD-*wildtype (WT) and heterozygous *DPYD*-Haplotype B3 status (mut). For all patients CTC-AEs were graded in each of the four CTx cycles across 14 areas. CTC-AE grading was performed according to the National-Cancer-Institute Common Terminology Criteria for Adverse Events, version 3.0 [17].

|  | **CTC-AE level** | **∑** | **%** | **WT+CTx** | **%** | **mut+CTx** | **%** |
| --- | --- | --- | --- | --- | --- | --- | --- |
| **Area** (defined) | **n** | **172** | **100.0** | **152** | **100.0** | **20** | **100.0** |
| hemoglobin | none | 61 | 36.7 | 57 | 37.7 | 4 | 26.7 |
|  | low | 105 | 63.3 | 94 | 62.3 | 11 | 73.3 |
|  | high | 0 | 0 | 0 | 0 | 0 | 0 |
|  | missing * | 6 | | 1 | | 5 | |
| leukocytes | none | 134 | 80.7 | 122 | 80.8 | 12 | 80.0 |
|  | low | 30 | 18.1 | 27 | 17.9 | 3 | 20.0 |
|  | high | 2 | 1.2 | 2 | 1.3 | 0 | 0 |
| neutrophiles | none | 154 | 92.8 | 139 | 92.1 | 15 | 100.0% |
|  | low | 10 | 6.0 | 10 | 6.6 | 0 | 0 |
|  | high | 2 | 1.2 | 2 | 1.3 | 0 | 0 |
| thrombocytes | none | 138 | 83.1 | 129 | 85.4 | 9 | 60.0 |
|  | low | 28 | 16.9 | 22 | 14.6 | 6 | 40.0 |
|  | high | 0 |  | 0 |  | 0 |  |
| alkaline phos-phastase | none | 147 | 88.6 | 134 | 88.7 | 13 | 86.7 |
|  | low | 19 | 11.4 | 17 | 11.3 | 2 | 13.3 |
|  | high | 0 |  | 0 |  | 0 |  |
| transaminases | none | 116 | 69.9 | 104 | 68.9 | 12 | 80.0 |
|  | low | 50 | 30.1 | 47 | 31.1 | 3 | 20.0 |
|  | high | 0 | 0 | 0 | 0 | 0 | 0 |
| creatinine | none | 143 | 86.1 | 132 | 87.4 | 11 | 73.3 |
|  | low | 23 | 13.9 | 19 | 12.6 | 4 | 26.7 |
|  | high | 0 | 0 | 0 | 0 | 0 | 0 |
| mucositis, stomatitis | none | 155 | 93.4 | 142 | 94.0 | 13 | 86.7 |
|  | low | 11 | 6.6 | 9 | 6.0 | 2 | 13.3 |
|  | high | 0 | 0 | 0 | 0 | 0 | 0 |
| nausea | none | 100 | 60.2 | 88 | 58.3 | 12 | 80.0 |
|  | low | 64 | 38.6 | 61 | 40.4 | 3 | 20.0 |
|  | high | 2 | 1.2 | 2 | 1.3 | 0 | 0 |
| vomoting | none | 151 | 91.0 | 136 | 90.1 | 15 | 100 |
|  | low | 14 | 8.4 | 14 | 9.3 | 0 | 0 |
|  | high | 1 | 0.6 | 1 | 0.7 | 0 | 0 |
| obstipation | none | 157 | 94.6 | 142 | 94.0 | 15 | 100.0 |
|  | low | 8 | 4.8 | 8 |  | 0 | 0 |
|  | high | 1 | 0.6 | 1 | 0.7 | 0 | 0 |
| diarrhea | none | 147 | 88.6 | 133 | 88.1 | 14 | 93.3 |
|  | low | 18 | 10.8 | 17 | 11.3 | 1 | 6.7 |
|  | high | 1 | 0.6 | 1 | 0.7 | 0 | 0 |
| sensorium | none | 60 | 36.1 | 58 | 38.4 | 2 | 13.3 |
|  | low | 104 | 62.7 | 91 | 60.3 | 13 | 86.7 |
|  | high | 2 | 1.2 | 2 | 1.3 | 0 | 0 |
|  | | | | | | | |
| **Area** (not defined) | **n** | **215** | **100.0** | **191** | **100.0** | **24** | **100.0** |
| other CTC-AEs | none | 92 | 44.4 | 84 | 44.7 | 8 | 42.1 |
|  | low | 105 | 50.7 | 96 | 51.1 | 9 | 47.4 |
|  | high | 10 | 4.8 | 8 | 4.3 | 2 | 10.5 |
|  | missing | 8 | | 3 | | 5 | |

**Table A.7: CTC-AEs (grouped) per defined area**

*: these missing values are given exemplarily for all the following areas (from leukocytes to sensorium); WT: wildtype; mut: patients with Haplotype B3 mutation; CTx: FOLFOX chemotherapy; CTC-AE grading was performed according to the National-Cancer-Institute Common Terminology Criteria for Adverse Events, version 3.0 [17].

|  | **CTC-AE level** | **∑** | **%** | **WT+CTx** | **%** | **mut+CTx** | **%** |
| --- | --- | --- | --- | --- | --- | --- | --- |
| **Area** (defined) | **n** | **43** | 100.0 | **38** | 100.0 | **5** | 100.0 |
| hemoglobin | none | 13 | 30.2 | 12 | 31.6 | 1 | 20.0 |
|  | low | 30 | 69.8 | 26 | 68.4 | 4 | 80.0 |
|  | high | 0 | 0 | 0 | 0 | 0 | 0 |
| leukocytes | none | 40 | 93.0 | 35 | 92.1 | 5 | 100.0 |
|  | low | 3 | 7.0 | 3 | 7.9 | 0 | 0 |
|  | high | 0 | 0 | 0 | 0 | 0 | 0 |
| neutrophiles | none | 42 | 97.7 | 37 | 97.4 | 5 | 100.0 |
|  | low | 1 | 2.3 | 1 | 2.6 | 0 | 0 |
|  | high | 0 | 0 | 0 | 0 | 0 | 0 |
| thrombocytes | none | 43 | 100.0 | 38 | 100.0 | 5 | 100.0 |
|  | low | 0 | 0 | 0 | 0 | 0 | 0 |
|  | high | 0 | 0 | 0 | 0 | 0 | 0 |
| alkaline phos-phastase | none | 39 | 90.7 | 35 | 92.1 | 4 | 80.0 |
|  | low | 4 | 9.3 | 3 | 7.9 | 1 | 20.0 |
|  | high | 0 | 0 | 0 | 0 | 0 | 0 |
| transaminases | none | 36 | 83.7 | 32 | 84.2 | 4 | 80.0 |
|  | low | 7 | 16.3 | 6 | 15.8 | 1 | 20.0 |
|  | high | 0 | 0 | 0 | 0 | 0 | 0 |
| creatinine | none | 37 | 86.0 | 34 | 89.5 | 3 | 60.0 |
|  | low | 6 | 14.0 | 4 | 10.5 | 2 | 40.0 |
|  | high | 0 | 0 | 0 | 0 | 0 | 0 |
| mucositis, stomatitis | none | 40 | 93.0 | 36 | 94.7 | 4 | 80.0 |
|  | low | 3 | 7.0 | 2 | 5.3 | 1 | 20.0 |
|  | high | 0 | 0 | 0 | 0 | 0 | 0 |
| nausea | none | 25 | 58.1 | 22 | 57.9 | 3 | 60.0 |
|  | low | 17 | 39.5 | 15 | 39.5 | 2 | 40.0 |
|  | high | 1 | 2.3 | 1 | 2.6 | 0 | 0.0 |
| vomoting | none | 38 | 88.4 | 33 | 86.8 | 5 | 100.00 |
|  | low | 4 | 9.3 | 4 | 10.5 | 0 | 0 |
|  | high | 1 | 2.3 | 1 | 2.6 | 0 | 0 |
| obstipation | none | 39 | 90.7 | 34 | 89.5 | 5 | 100.0 |
|  | low | 4 | 9.3 | 4 | 10.5 | 0 | 0 |
|  | high | 0 | 0 | 0 | 0 | 0 | 0 |
| diarrhea | none | 39 | 90.7 | 35 | 92.1 | 4 | 80.0 |
|  | low | 4 | 9.3 | 3 | 7.9 | 1 | 20.0 |
|  | high | 0 | 0 | 0 | 0 | 0 | 0 |
| sensorium | none | 28 | 65.1 | 26 | 68.4 | 2 | 40.0 |
|  | low | 14 | 32.6 | 11 | 28.9 | 3 | 60.0 |
|  | high | 1 | 2.3 | 1 | 2.6 | 0 | 0 |
|  | | | | | | | |
| **Area** (not defined) | **n** | **51** | **100.0** | **42** | **100.0** | **9** | **100.0** |
| other CTC-AEs | none | 28 | 54.9 | 25 | 59.5 | 3 | 33.3 |
|  | low | 20 | 39.2 | 16 | 38.1 | 4 | 44.4 |
|  | high | 3 | 5.9 | 1 | 2.4 | 2 | 22.2 |

**Table A.8: Differences between groups per area in the first CTx-cycle**

*: Studentized permutation test; WT: wildtype; mut: patients with Haplotype B3 mutation; CTx: FOLFOX chemotherapy. CTC-AE grading was performed according to the National-Cancer-Institute Common Terminology Criteria for Adverse Events, version 3.0 [17].

|  | **CTC-AE level** | **∑** | **%** | **1^st^ cycle** | **%** | **2^nd^ cycle** | **%** | **3^rd^  cycle** | **%** | **4^th^  cycle** | **%** |
| --- | --- | --- | --- | --- | --- | --- | --- | --- | --- | --- | --- |
| **Area** (defined) | **n** |  |  | **43** | 100.0 | **43** | 100.0 | **43** | 100.0 | **43** | 100.0 |
| hemoglobin | none | 61 | 36.7 | 13 | 30.2 | 18 | 42.9 | 16 | 39.0 | 14 | 35.0 |
|  | low | 105 | 63.3 | 30 | 69.8 | 24 | 57.1 | 25 | 61.0 | 26 | 65.0 |
|  | high | 0 | 0 | 0 | 0 | 0 | 0 | 0 | 0 | 0 | 0 |
|  | missing * | 6 | | 0 | | 1 | | 2 | | 3 | |
| leukocytes | none | 134 | 80.7 | 40 | 93.0 | 33 | 78.6 | 33 | 80.5 | 28 | 70.0 |
|  | low | 30 | 18.1 | 3 | 7.0 | 8 | 19.0 | 7 | 17.1 | 12 | 30.0 |
|  | high | 2 | 1.2 | 0 | 0 | 1 | 2.4 | 1 | 2.4 | 0 | 0 |
| neutrophiles | none | 154 | 92.8 | 42 | 97.7 | 39 | 92.9 | 38 | 92.7 | 35 | 87.5 |
|  | low | 10 | 6.0 | 1 | 2.3 | 1 | 2.4 | 3 | 7.3 | 5 | 12.5 |
|  | high | 2 | 1.2 | 0 | 0 | 2 | 4.8 | 0 | 0 | 0 | 0 |
| thrombocytes | none | 138 | 83.1 | 43 | 100.0 | 36 | 85.7 | 29 | 70.7 | 30 | 75.0 |
|  | low | 28 | 16.9 | 0 | 0 | 6 | 14.3 | 12 | 29.3 | 10 | 25.0 |
|  | high | 0 | 0 | 0 | 0 | 0 | 0 | 0 | 0 | 0 | 0 |
| alkaline phos-phastase | none | 147 | 88.6 | 39 | 90.7 | 38 | 90.5 | 36 | 87.8 | 34 | 85.0 |
|  | low | 19 | 11.4 | 4 | 9.3 | 4 | 9.5 | 5 | 12.2 | 6 | 15.0 |
|  | high | 0 | 0 | 0 | 0 | 0 | 0 | 0 | 0 | 0 | 0 |
| transaminases | none | 116 | 69.9 | 36 | 83.7 | 30 | 71.4 | 26 | 63.4 | 24 | 60.0 |
|  | low | 50 | 30.1 | 7 | 16.3 | 12 | 28.6 | 15 | 36.6 | 16 | 40.0 |
|  | high | 0 | 0 | 0 | 0 | 0 | 0 | 0 | 0 | 0 | 0 |
| creatinine | none | 143 | 86.1 | 37 | 86.0 | 35 | 83.3 | 35 | 85.4 | 36 | 90.0 |
|  | low | 23 | 13.9 | 6 | 14.0 | 7 | 16.7 | 6 | 14.6 | 4 | 10.0 |
|  | high | 0 | 0 | 0 | 0 | 0 | 0 | 0 | 0 | 0 | 0 |
| mucositis, stomatitis | none | 155 | 93.4 | 40 | 93.0 | 38 | 90.5 | 40 | 97.6 | 37 | 92.5 |
|  | low | 11 | 6.6 | 3 | 7.0 | 4 | 9.5 | 1 | 2.4 | 3 | 7.5 |
|  | high | 0 | 0 | 0 | 0 | 0 | 0 | 0 | 0 | 0 | 0 |
| nausea | none | 100 | 60.2 | 25 | 58.1 | 26 | 61.9 | 25 | 61.0 | 24 | 60.0 |
|  | low | 64 | 38.6 | 17 | 39.5 | 15 | 35.7 | 16 | 39.0 | 16 | 40.0 |
|  | high | 2 | 1.2 | 1 | 2.3 | 1 | 2.4 | 0 | 0 | 0 | 0 |
| vomoting | none | 151 | 91.0 | 38 | 88.4 | 37 | 88.1 | 39 | 95.1 | 37 | 92.5 |
|  | low | 14 | 8.4 | 4 | 9.3 | 5 | 11.9 | 2 | 4.9 | 3 | 7.5 |
|  | high | 1 | 0.6 | 1 | 2.3 | 0 | 0 | 0 | 0 | 0 | 0 |
| obstipation | none | 157 | 94.6 | 39 | 90.7 | 41 | 97.6 | 40 | 97.6 | 37 | 92.5 |
|  | low | 8 | 4.8 | 4 | 9.3 | 0 | 0 | 1 | 2.4 | 3 | 7.5 |
|  | high | 1 | 0.6 | 0 | 0 | 1 | 2.4 | 0 | 0 | 0 | 0 |
| diarrhea | none | 147 | 88.6 | 39 | 90.7 | 38 | 90.5 | 36 | 87.8 | 34 | 85.0 |
|  | low | 18 | 10.8 | 4 | 9.3 | 4 | 9.5 | 5 | 12.2 | 5 | 12.5 |
|  | high | 1 | 0.6 | 0 | 0 | 0 | 0 | 0 | 0 | 1 | 2.5 |
| sensorium | none | 60 | 36.1 | 28 | 65.1 | 16 | 38.1 | 8 | 19.5 | 8 | 20.0 |
|  | low | 104 | 62.7 | 14 | 32.6 | 26 | 61.9 | 32 | 78.0 | 32 | 80.0 |
|  | high | 2 | 1.2 | 1 | 2.3 | 0 | 0 | 1 | 2.4 | 0 | 0 |
|  | | | | | | | | | | | |
| **Area** (not defined) | **n** |  |  | **51** | **100.0** | **54** | **100.0** | **58** | **100.0** | **52** | **100.0** |
| other CTC-AEs | none | 92 | 44.4 | 28 | 54.9 | 25 | 47.2 | 20 | 35.7 | 19 | 40.4 |
|  | low | 105 | 50.7 | 20 | 39.2 | 28 | 52.8 | 31 | 55.4 | 26 | 55.3 |
|  | high | 10 | 4.8 | 3 | 5.9 | 0 | 0 | 5 | 8.9 | 2 | 4.3 |
|  | missing | 8 | | 0 | | 1 | | 2 | | 5 | |

**Table A.9: Differences between CTx cycles per area in all patients**

*: these missing values are given exemplarily for all the following areas (from leukocytes to senso­rium); CTC-AE levels: grading (G) of the adverse events according to NCI-CTC-AE-criteria in G0 (none), in G1 - 2 (low) and ≥ G3 (high). No G5 (death) had been observed. CTC-AE grading was used according to the National-Cancer-Institute Common Terminology Criteria for Adverse Events, version 3.0 [17].

|  |  |  |  | **Postsurgical FOLFOX chemotherapy (CTx)** | | | | | | | |
| --- | --- | --- | --- | --- | --- | --- | --- | --- | --- | --- | --- |
|  | **CTC-AE level** | **∑** | **%** | **1st cycle** | **%** | **2nd cycle** | **%** | **3rd  cycle** | **%** | **4th  cycle** | **%** |
| **Area** (defined) | **n** |  |  | **5** | 100.0 | **5** | 100.0 | **5** | 100.0 | **5** | 100.0 |
| hemoglobin | none | 4 | 26.7 | 1 | 20.0 | 1 | 25.0 | 1 | 33.3 | 1 | 33.3 |
|  | low | 11 | 73.3 | 4 | 80.0 | 3 | 75.0 | 2 | 66.7 | 2 | 66.7 |
|  | high | 0 | 0 | 0 | 0 | 0 | 0 | 0 | 0 | 0 | 0 |
|  | missing * | 5 | | 0 | | 1 | | 2 | | 2 | |
| leukocytes | none | 12 | 80.0 | 5 | 100.0 | 3 | 75.0 | 2 | 66.7 | 2 | 66.7 |
|  | low | 3 | 20.0 | 0 | 0 | 1 | 25.0 | 1 | 33.3 | 1 | 33.3 |
|  | high | 0 | 0 | 0 | 0 | 0 | 0 | 0 | 0 | 0 | 0 |
| neutrophiles | none | 15 | 100.0 | 5 | 100.0 | 4 | 100.0 | 3 | 100.0 | 3 | 100.0 |
|  | low | 0 | 0 | 0 | 0 | 0 | 0 | 0 | 0 | 0 | 0 |
|  | high | 0 | 0 | 0 | 0 | 0 | 0 | 0 | 0 | 0 | 0 |
| thrombocytes | none | 9 | 60.0 | 5 | 100.0 | 2 | 50.0 | 1 | 33.3 | 1 | 33.3 |
|  | low | 6 | 40.0 | 0 | 0 | 2 | 50.0 | 2 | 66.7 | 2 | 66.7 |
|  | high | 0 | 0 | 0 | 0 | 0 | 0 | 0 | 0 | 0 | 0 |
| alkaline phos-phastase | none | 13 | 86.7 | 4 | 80.0 | 4 | 100.0 | 2 | 66.7 | 3 | 100.0 |
|  | low | 2 | 13.3 | 1 | 20.0 | 0 | 0 | 1 | 33.3 | 0 | 0 |
|  | high | 0 | 0 | 0 | 0 | 0 | 0 | 0 | 0 | 0 | 0 |
| transaminases | none | 12 | 80.0 | 4 | 80.0 | 4 | 100.0 | 2 | 66.7 | 2 | 66.7 |
|  | low | 3 | 20.0 | 1 | 20.0 | 0 | 0 | 1 | 33.3 | 1 | 33.3 |
|  | high | 0 | 0 | 0 | 0 | 0 | 0 | 0 | 0 | 0 | 0 |
| creatinine | none | 11 | 73.3 | 3 | 60.0 | 3 | 75.0 | 2 | 66.7 | 3 | 100.0 |
|  | low | 4 | 26.7 | 2 | 40.0 | 1 | 25.0 | 1 | 33.3 | 0 | 0 |
|  | high | 0 | 0 | 0 | 0 | 0 | 0 | 0 | 0 | 0 | 0 |
| mucositis, stomatitis | none | 13 | 86.7 | 4 | 80.0 | 4 | 100.0 | 3 | 100.0 | 2 | 66.7 |
|  | low | 2 | 13.3 | 1 | 20.0 | 0 | 0 | 0 | 0 | 1 | 33.3 |
|  | high | 0 | 0 | 0 | 0 | 0 | 0 | 0 | 0 | 0 | 0 |
| nausea | none | 12 | 80.0 | 3 | 60.0 | 3 | 75.0 | 3 | 100.0 | 3 | 100.0 |
|  | low | 3 | 20.0 | 2 | 40.0 | 1 | 25.0 | 0 | 0 | 0 | 0 |
|  | high | 0 | 0 | 0 | 0 | 0 | 0 | 0 | 0 | 0 | 0 |
| vomoting | none | 15 | 100.0 | 5 | 100.0 | 4 | 100.0 | 3 | 100.0 | 3 | 100.0 |
|  | low | 0 | 0 | 0 | 0 | 0 | 0 | 0 | 0 | 0 | 0 |
|  | high | 0 | 0 | 0 | 0 | 0 | 0 | 0 | 0 | 0 | 0 |
| obstipation | none | 15 | 100.0 | 5 | 100.0 | 4 | 100.0 | 3 | 100.0 | 3 | 100.0 |
|  | low | 0 | 0 | 0 | 0 | 0 | 0 | 0 | 0 | 0 | 0 |
|  | high | 0 | 0 | 0 | 0 | 0 | 0 | 0 | 0 | 0 | 0 |
| diarrhea | none | 14 | 93.3 | 4 | 80.0 | 4 | 100.0 | 3 | 100.0 | 3 | 100.0 |
|  | low | 1 | 6.7 | 1 | 20.0 | 0 | 0 | 0 | 0 | 0 | 0 |
|  | high | 0 | 0 | 0 | 0 | 0 | 0 | 0 | 0 | 0 | 0 |
| sensorium | none | 2 | 13.3 | 2 | 40.0 | 0 | 0 | 0 | 0 | 0 | 0 |
|  | low | 13 | 86.7 | 3 | 60.0 | 4 | 100.0 | 3 | 100.0 | 3 | 100.0 |
|  | high | 0 | 0 | 0 | 0 | 0 | 0 | 0 | 0 | 0 | 0 |
|  | | | | | | | | | | | |
| **Area** (not defined) | **n** |  |  | **9** | **100.0** | **5** | **100.0** | **5** | **100.0** | **5** | **100.0** |
| other CTC-AEs | none | 8 | 42.1 | 3 | 33.3 | 2 | 50.0 | 2 | 66.7 | 1 | 33.3 |
|  | low | 9 | 47.4 | 4 | 44.4 | 2 | 50.0 | 1 | 33.3 | 2 | 66.7 |
|  | high | 2 | 10.5 | 2 | 22.2 | 0 | 0 | 0 | 0 | 0 | 0 |
|  | missing | 5 | | 0 | | 1 | | 2 | | 2 | |

**Table A.10: Differences between CTx cycles per area in het_HapB3-patients**

*: these missing values are given exemplarily for all the following areas (from leukocytes to sen­sorium); CTC-AE levels: grading (G) of the adverse events in G0 (none), in G1 - 2 (low) and ≥ G3 (high). No G5 (death) had been observed. CTC-AE grading was performed according to the National-Cancer-Institute Common Terminology Criteria for Adverse Events, version 3.0 [17].

|  | **CTC-AE_class** | | |
| --- | --- | --- | --- |
| **Predictors** | **Odds Ratios** | **CI** | **p-value** |
| (Intercept) | 0.18 | 0.13 – 0.26 | **<0.001** |
| group [mut+FOLFOX] | 1.84 | 0.77 – 4.37 | 0.170 |
| N _study_patient number_ | 39 | | |
| **Observations** | 554 | | |
| **Marginal R^2^ / conditional R^2^** | 0.011 / 0.129 | | |

**Table A.11: Risk for any-grade CTC-AEs at CTx cycle 1**

No significantly increased risk for any grades of CTx-associated AEs was found in patients with het_*DPYD*-Haplotype B3 (mut) compared to *DPYD*-WT during the first FOLFOX cycle. The table shows regression coefficients (as odds ratios, ORs) from a mixed effect logistic regression model for high-grade toxicity on CTC-AEs assessed in 14 areas. CTC-AE grading was used according to the National-Cancer-Institute Common Terminology Criteria for Adverse Events, version 3.0 [17].

|  | **CTC-AE_class** | | |
| --- | --- | --- | --- |
| **Predictors** | **Odds Ratios** | **CI** | **p-value** |
| (Intercept) | 0.28 | 0.21 – 0.38 | **<0.001** |
| CTx cycle [3^rd^ cycle] | 1.28 | 0.97 – 1.68 | 0.076 |
| CTx cycle [4^th^ cycle] | 1.33 | 1.02 – 1.75 | **0.038** |
| group [mut+FOLFOX] | 0.93 | 0.41 – 2.14 | 0.873 |
| Δ relative applied 5-FU dosage | 2.67 | 1.00 – 7.12 | **0.051** |
| N _study_patient number_ | 42 | | |
| **Observations** | 1755 | | |
| **Marginal R^2^ / conditional R^2^** | 0.008 / 0.129 | | |

**Table A.12: Risk for any-grade CTC-AEs at CTx cycle 2 to 4**

Compared to CTx cycle 2 there was a higher chance for patients to suffer AEs at later cycles: [at 3^rd^ CTx cycle: OR 1.28; p=0.076; at 4^th^ cycle: OR 1.33; p=0.038]. 5-FU dose reduction from previous cycle shows an impact on the occurrence of CTx-associated CTC-AEs [OR 2.67; p=0.051]. No difference was observed between patients with *DPYD*-WT- and het_HapB3-status [OR 0.93; p=0.873]. The table shows regression coefficients (as odds ratios, ORs) from a mixed effect logistic regression model.

| **Patients** | **5-FU dose reduction** | **no dose reduction** | **∑** |
| --- | --- | --- | --- |
| group with mut + FOLFOX | 4 | 1 | 5 |
| group with FOLFOX | 13 | 25 | 38 |
| **∑** | 17 | 26 | 43 |

**Table A.13: Influence of *DPYD* mutation on 5-FU dose reduction**

As shown in the cross tabulation, 26 (60.5 %) study participants had received FOLFOX-CTx without dose-reduction (25 patients with *DPYD*-WT and 1 patient with het_HapB3-status).

|  | **CTx dose reduction*  (yes = 1 versus no = 0)** | | |
| --- | --- | --- | --- |
| **Predictors** | **Odds Ratios** | **CI** | **p-value** |
| (Intercept) | 0.05 | 0.00 – 4.96 | 0.222 |
| group [mut + FOLFOX] | 12.55 | 1.38 – 289.22 | **0.044** |
| sex [male] | 0.23 | 0.05 – 0.95 | **0.049** |
| age at date of surgery | 1.05 | 0.98 – 1.14 | 0.188 |
| **Observations** | 43 | | |
| **R^2^ Tjur** | 0.216 | | |

**Table A.14: Modeling of CTx dose-reduction on the level of patients**

*All data are given for 5-FU; evaluation of mutational impact on 5-FU dose-reduction, R2 Tjur: Tjur’s coefficient of discrimination. Patients with het_HapB3-status had a higher chance to experience dose-reductions (OR=12.55; p=0.044)

|  | **CTx dose reduction*  (yes = 1 versus no = 0)** | | |
| --- | --- | --- | --- |
| **Predictors** | **Odds Ratios** | **CI** | **p-value** |
| (Intercept) | 0.00 | 0.00 – 0.09 | **0.020** |
| grouped [mut + FOLFOX] | 812.47 | 0.91 – 723017.68 | 0.053 |
| Sex [male] | 0.07 | 0.00 – 2.51 | 0.143 |
| Age at date of surgery | 1.16 | 0.95 – 1.14 | 0.138 |
| **Observations** | 1757 | | |
| **Marginal R^2^ / conditional R^2^** | 0.184 / 0.915 | | |

**Table A.15: Prediction of dose-reduction on the level of CTx cycles**

| **Parameter** | **level** | **WT + CTx** | **mut + CTx** | **p-value** | **test** |
| --- | --- | --- | --- | --- | --- |
| **n (events)** |  | 38 | 5 |  |  |
| **relative 5-FU dose applied** (in mean) |  |  |  | 0.13 | Studentized permu- tation test |
|  | mean ± SD | 0.93 ± 0.12 | 0.69 ± 0.35 |  |  |
|  | median | 1.0 | 0.88 |  |  |
|  | (min; max) | 0.55; 1.0 | 0.24; 1.0 |  |  |
|  |  |  |  |  |  |
| **relative 5-FU dose applied** (cut-off 75%) |  |  |  | 0.06 | Fisher`s exact test for count data |
|  | < 75% | 2 (5.3%) | 2 (40.0%) |  |  |
|  | ≥ 75% | 36 (94.7%) | 3 (60.0%) |  |  |

**Table A.16: Prediction of the amount of 5-FU dose-reduction on the level of patients**

| **Term** | **HR** | **CI** | **p-value** |
| --- | --- | --- | --- |
| age at date of surgery, 12 months | 1.054 | [0.99 ; 1.1] | 0.106 |
| sex [male] | 2.276 | [0.68 ; 7.6] | 0.183 |
| group [mut + FOLFOX] | 3.774 | [0.96 ; 14.8] | **0.057** |
| relative 5-FU dose (100% as planned) | 1.05 | [0.26 ; 2.5] | 0.708 |

**Table A.17: Model coefficients from the COX model for DFS**

HR: hazard ratio; CI: 95% confidence interval; COX model for disease-free survival (DFS).


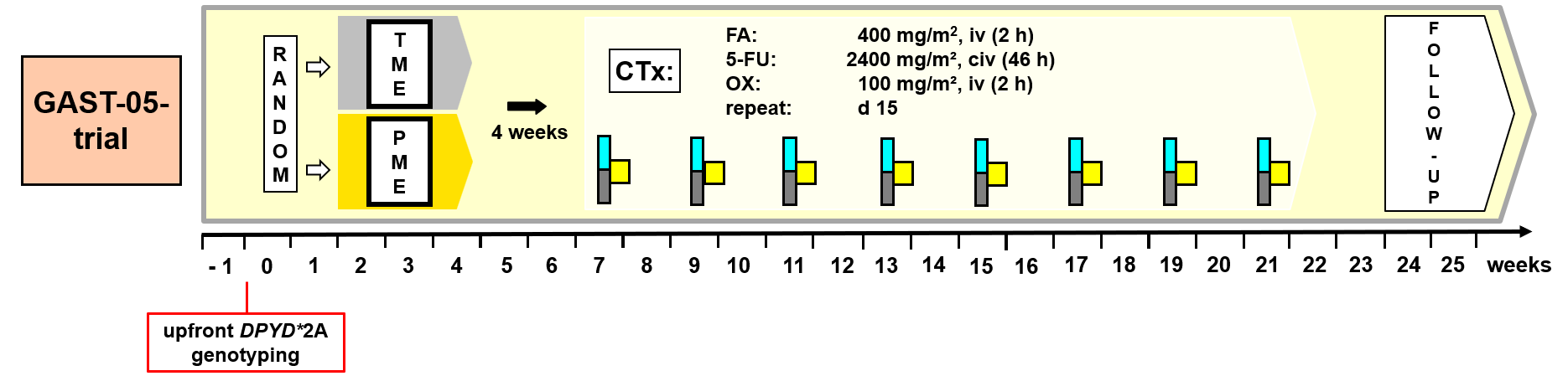


**Figure A.1: GAST-05-trial** **design**

All participants of the GAST-05-trial (ISRCTN35198481) with locally advanced adenocarcinomas in the upper rectum (cUICC stages ≥ II, inferior tumor margin ≥12 cm above the anal verge) had been treated either by total (TME) or partial (PME) mesorectal excision followed by 8 applications (4 cycles) of modified FOLFOX chemotherapy (CTx). FA: folinic acid; 5-FU: 5-Fluorouracil; OX: oxaliplatin; iv: intravenous application; civ: conti­nuous intravenous application. For all patients upfront genotyping for *DPYD**2A mutations was mandatory at study entry. During treatment, patients were monitored twice (or more) per week, with appropriate adjustments of the CTx made as necessary. Peri- and postoperative complications as well as CTx-associated adverse events (AEs) were assessed according to established classification systems. All patients were followed at three-months intervals for two years and then at six-months intervals for >3 years. Evaluations consisted of physical examination, complete blood cell counts, and blood chemistry analyses. Procto-/rectoscopy, abdominal ultrasound, computed tomography (CT) of the thorax, abdomen and pelvis, and conventional X-ray of the chest were conducted according to the GAST-05-trial protocol. Histopathological confirmation of local recurrence (defined as cancer relapse within 5 cm above or below the anastomotic height) and of distant cancer recurrence was expressly requested; in exceptional cases alternative approaches included sequential radiologic examinations to detect the progression of a mass suspicious for cancer relapse. The GAST-05-trial was funded by the German Research Foundation (DFG; BE 3649/3-1) and conducted by the principle investigators Heinz Becker, MD (*in memoriam,* deceased 09/2014), and Torsten Liersch, MD.


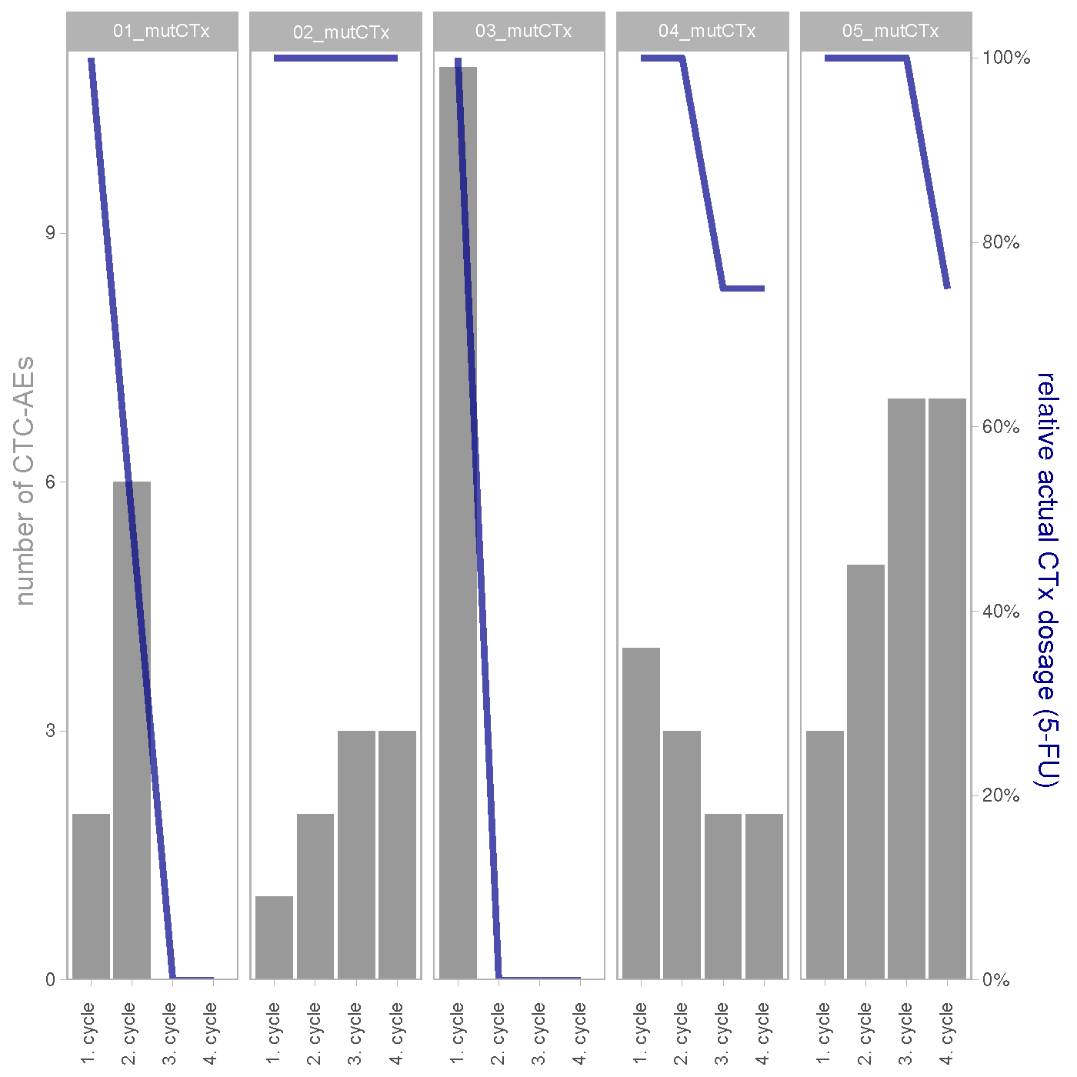


**Figure A2: Effects of clinical monitoring in patients with het_*DPYD*-Haplotype B3**

The number of CTC-AEs (gray colored) per FOLFOX-cycle is shown for those 5 patients in whom a *DPYD-*Haplotype B3 mutation has been newly detected. The blue line represents the respective relative 5-FU dose as applied at the respective time point. The figure demonstrates the effects of daily clinical monitoring (following the GAST-05 trial protocol). CTC-AE: NCI-Common Toxicity Criteria of Adverse Events, version 3.0 [17].


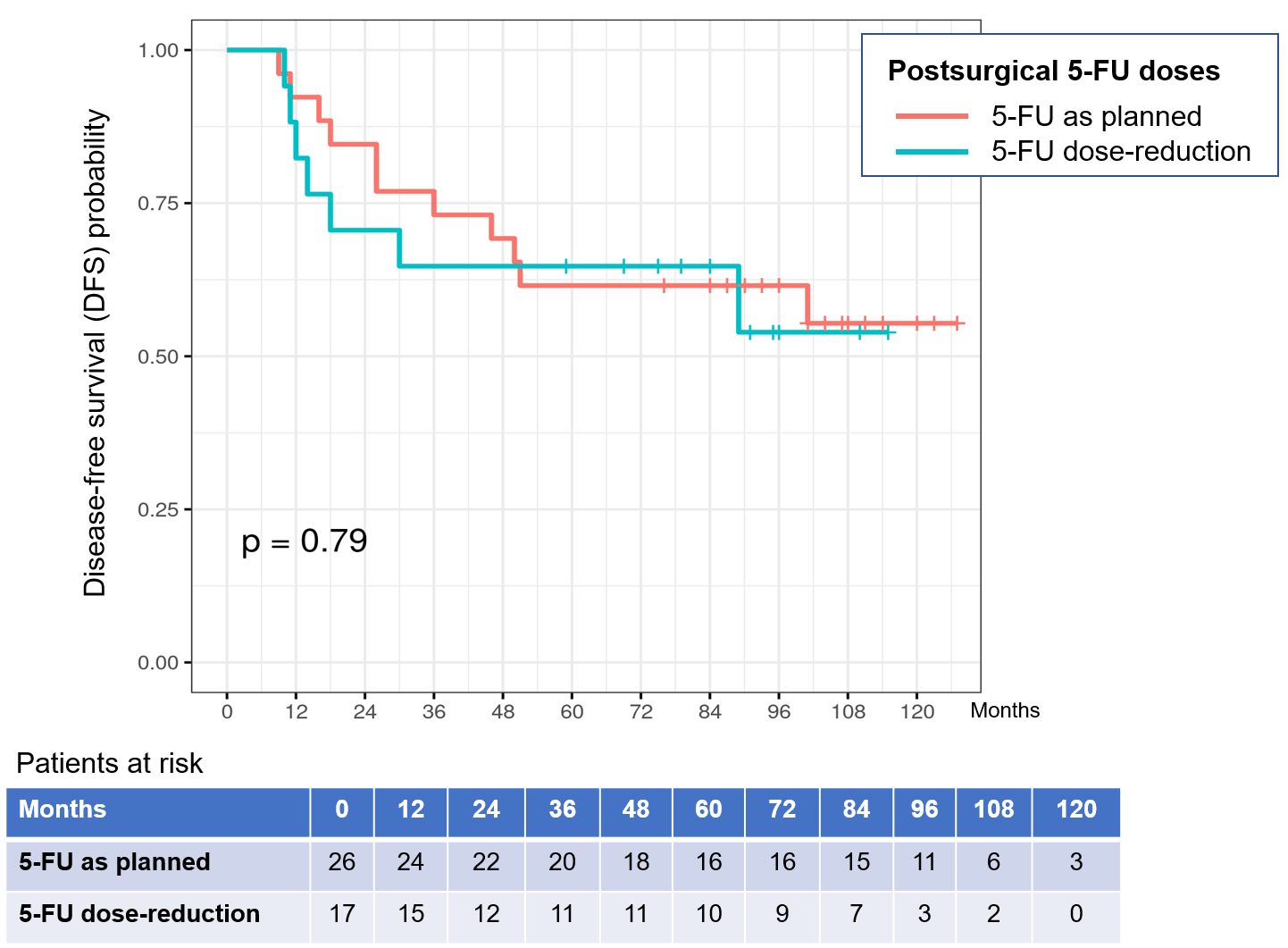


**Figure A.3: 5-FU dose reduction (any amount) and DFS**

The Kaplan-Meier estimator demonstrates the disease-free survival (DFS) probability for patients without any 5-FU dose reduction (red line) during postsurgical FOLFOX CTx. Blue line: patients with 5-FU dose reduction during postsurgical treatment; logrank test: p=0.79.
